# A Combined CNN-LSTM Network for Ship Classification on SAR Images

**DOI:** 10.3390/s24247954

**Published:** 2024-12-12

**Authors:** Abdelmalek Toumi, Jean-Christophe Cexus, Ali Khenchaf, Mahdi Abid

**Affiliations:** ENSTA Bretagne, Lab-STICC, UMR CNRS 6285, 29806 Brest, France; jean-christophe.cexus@ensta-bretagne.fr (J.-C.C.); ali.khenchaf@ensta-bretagne.fr (A.K.);

**Keywords:** SAR imagery, ships classification, deep learning, convolutional neural networks, synthetic aperture radar (SAR)

## Abstract

Satellite SAR (synthetic aperture radar) imagery offers global coverage and all-weather recording capabilities, making it valuable for applications like remote sensing and maritime surveillance. However, its use in machine learning-based automatic target classification faces challenges, including the limited availability of SAR target training samples and the inherent constraints of SAR images, which provide less detailed features compared to natural images. These issues hinder the effective training of convolutional neural networks (CNNs) and complicate the transfer learning process due to the distinct imaging mechanisms of SAR and natural images. To address these challenges, we propose a shallow CNN architecture specifically designed to optimize performance on SAR datasets. Evaluations were performed on three datasets: FUSAR-Ship, OpenSARShip, and MSTAR. While the FUSAR-Ship and OpenSARShip datasets present difficulties due to their limited and imbalanced class distributions, MSTAR serves as a benchmark with balanced classes. To compare and optimize the proposed shallow architecture, we examine various properties of CNN components, such as the filter numbers and sizes in the convolution layers, to reduce redundancy, improve discrimination capability, and decrease network size and learning time. In the second phase of this paper, we combine the CNN with Long short-term memory (LSTM) networks to enhance SAR image classification. Comparative experiments with six state-of-the-art CNN architectures (VGG16, ResNet50, Xception, DenseNet121, EfficientNetB0, and MobileNetV2) demonstrate the superiority of the proposed approach, achieving competitive accuracy while significantly reducing training times and network complexity. This study underscores the potential of customized architectures to address SAR-specific challenges and enhance the efficiency of target classification.

## 1. Introduction

Synthetic aperture radar (SAR) technology has revolutionized maritime surveillance and object and vessel classification by providing high-resolution, all-weather imaging capabilities. SAR sensors have become instrumental in monitoring vast maritime areas and in ship detection, identification, and classification. In recent years, significant advancements have been made in the field of ship and object classification using SAR imagery, enhancing maritime security, search-and-rescue operations, marine transportation management, marine security situational awareness, environmental monitoring, and so on.

With the rapid development of satellite imagery, the need to analyze these images grows more every day. A promising solution is the use of artificial intelligence to extract and classify the different targets found in satellite images. In the case of vehicles, artificial intelligence (AI) can help recognize models and determine specific features such as the speed of movement or the dimensions of the targets. Multiple techniques and algorithms have been developed to find better solutions, leading to the use of deep neural networks. By adapting deep neural networks specialized in object detection and identification, we can now recognize specific ships and vehicles using satellite imagery.

Recently, with advances in computational power and the ability to parallelize calculations using GPUs, DL architectures as instances of ML algorithms have been further investigated. Unlike traditional machine learning methods, deep neural networks (DNNs) mimic the functioning of the human brain and are parametric, meaning the number of their parameters is independent of the size of the training dataset. This feature is crucial during the prediction phase, as it reduces processing time and enhances the applicability of these algorithms for real-time image processing. Additional interest in using DL schemes arises from their rapid implementation on FPGAs [1] and ASICs [2].

DL has made great progress in a variety of real-world problems, e.g., detection, recognition, identification, motion tracking, action recognition, prediction, and data denoising or dehazing. In this context, we can mention CNNs, the “Boltzmann family” including deep belief networks, deep Boltzmann machines, and stacked auto-encoders (for denoising). Furthermore, we can also refer to recurrent neural networks (RNNs), which are more adapted for signals processed over variable observation windows [3]. In RNNs, long short-term memory (LSTM) networks [4] are usually used to explore temporal aspects and correlation in sequential and multi-view data [5].

Nevertheless, DL still has some limitations in generalizing performances and optimization architecture components in real-world applications that need to be studied in depth by researchers. One challenge is the insufficient number of SAR ship training samples, which hinders the effective training of CNNs. Additionally, the limited information available in SAR images, compared to natural images like those in ImageNet, restricts the extraction of discriminative feature descriptors. To overcome the problem of insufficient and unbalanced data, one of the most suitable strategies is to pre-train models from large datasets and adapt these models by transfer learning and fine-tuning on the target dataset (i.e., a SAR images dataset) [6,7]. The second strategy deals with online and offline data augmentation [8,9,10] and investigation on semi-supervised and unsupervised learning methods to deal with limited labeled SAR data [11]. Techniques like self-training and clustering-based approaches aim to enhance classification performance with minimal labeled samples.

On the other hand, to extract the most discriminative representation features from SAR images for target objects (such as ships and vehicles), extensive research has been conducted on various aspects, including network architecture design and optimization [12], embedding attention mechanisms [13,14,15], feature and/or decision fusion [16,17], learning strategies [18], one-shot learning [16,19], and more. Hence, efforts have been made to improve the performance of SAR object classification. However, these usually require more complex network structures, higher-dimensional features, and more costly storage costs.

Furthermore, a limited SAR dataset proves to be insufficient for adequately learning numerous parameters within a complex CNN. This inadequacy leads to overfitting the features of the ship extracted by CNN exhibiting significant redundancy, directly compromising the models’ discriminative capabilities [20]. In [21], many studies have explored CNN architectures for ship classification, leveraging their ability to automatically learn features from SAR images. These architectures are often fine-tuned or adapted to suit the unique characteristics of SAR data, achieving improved classification accuracy.

The authors in [22] provide a comprehensive overview of recent advances in ship detection and classification using deep learning models, focusing on SAR imagery. They review various architectures, including CNNs and detection models, and discuss challenges and future directions in the field. In ship classification, the complex nature of SAR data and the variability of ship signatures pose significant challenges for traditional classification methods. Existing approaches often struggle to effectively capture both the spatial and temporal characteristics of SAR images without including the sequential and temporal information.

To address these limitations, this paper proposes a novel deep learning architecture that combines shallow convolutional neural networks (CNNs) and long short-term memory (LSTM) for ship classification on limited datasets. By leveraging the strengths of both CNNs and LSTMs, our model effectively captures both local spatial features and temporal dependencies within limited SAR images, leading to significant improvements in classification accuracy compared to state-of-the-art methods.

In this context, several literature works are presented combining LSTM networks with CNN frameworks to enhance target detection and recognition in remote sensing data. The authors in [23] introduce an architecture known as Multi-Stream CNN (MS-CNN) for automatic target recognition (ATR) in synthetic aperture radar (SAR) by utilizing SAR images from various perspectives. They specifically implement a multi-input architecture that combines information from different views of the same target from diverse angles, allowing the innovative multi-view design of MS-CNN to maximize the utility of limited SAR image data and boost recognition performance. The authors provide a comprehensive overview of the literature on LSTMs that are specifically applied to multi-view ATR methods. To reduce the influence of azimuth variation on SAR ATR and extract azimuth-robust features from SAR series, the authors in [24] propose a Conv-BiLSTM Prototypical Network (CBLPN), which uses as the feature extractor a convolutional bidirectional long short-term memory (Conv-BiLSTM) adapted for few training samples. For classification, the authors propose a classifier based on Euclidean distance for few training samples.

Target detection in maritime radar data often struggles with issues such as clutter and low signal-to-noise ratios. To overcome these limitations, the work in [25] proposes a novel CNN-LSTM architecture specifically designed for augmenting target detection in real maritime wide-area surveillance radar data.

Some recent works explore the fusion of SAR imagery with other modalities, such as optical imagery or AIS (automatic identification system) data, using deep learning techniques. These multi-modal fusion methods [10] enhance the classification performance by leveraging complementary information from different sources. New techniques are being developed to enhance the ability to differentiate between features or to improve the performance of networks. They include attention mechanisms such as RasNet architecture [13] and Transformer-based architectures [26]. These architectures have gained traction for ship classification from SAR imagery. These mechanisms allow the model to focus on relevant regions in the SAR image, improving its ability to capture intricate ship features and aiding in accurate classification. In the same context, addressing the variability of spatiotemporal resolutions in SAR images, the RSMamba method is proposed [27]. RsMamba proposes an innovative architecture for remote sensing image classification. This approach leverages the State Space Model (SSM) framework alongside the hardware-efficient Mamba design [28], effectively combining a global receptive field with linear modeling complexity to deliver both efficiency and accuracy in classification tasks.

Researchers are also investigating semi-supervised and unsupervised learning methods [29] to deal with limited labeled SAR data. Techniques like self-training and clustering-based approaches aim to enhance classification performance with minimal labeled samples.

In the literature, the hybridization of CNNs and LSTM has been proposed across various application domains. For trajectory prediction, CNN-LSTM hybrid models [30] have been widely applied to aircraft 4D trajectory modeling and human-driven vehicle path forecasting, demonstrating their efficiency in handling spatial–temporal complexities. In machine vision and fuel consumption, the authors in [31] propose CNN-LSTM frameworks adapted for tasks such as feature extraction and prediction, improving accuracy and robustness. A comprehensive overview for advancements in deep learning techniques and the combination of CNN-LSTM in maritime applications can be found in [32]. In the medical field, particularly for COVID-19 detection and analysis, the study [5] introduces a hybrid CNN-LSTM approach to classify COVID-19 cases using sequential and temporal chest X-ray (CXR) images.

Our main contributions can be summarized as follows:

1. Specific focus on single-view SAR imagery: Unlike other studies, our research targets the challenges of synthetic aperture radar (SAR) imagery, such as limited labeled datasets, imbalanced class distributions, and non-sequential images.
2. Proposed optimizations: We propose a shallow CNN combined with LSTM to reduce network complexity, minimize training time, and improve classification accuracy for SAR datasets. This contrasts with standard CNN-LSTM implementations, which often prioritize depth and complexity. Through a systematic evaluation of CNN components, such as the number and size of filters, we aim to optimize the model’s performance while minimizing computational cost and training time.
3. Comprehensive validation: We validated our architecture on three distinct SAR datasets (FUSAR-Ship, OpenSARShip, and MSTAR), showcasing its adaptability and competitive performance in handling datasets of varying size, balance, and difficulty.

As part of this paper, we present a brief concept, the dataset, and the theoretical description of the proposed architectures. Then, we summarize the implementation of the different algorithms and the choices and solutions taken to circumvent the obstacles encountered. Finally, we evaluate and compare the proposed architectures with the results of classical convolutional neural networks.

## 2. Convolutional Neural Networks (CNNs)

### 2.1. Description of CNN

The principle consists of extracting the relevant features in an automatic way and carrying out the classification or identification phase. In this paper, we are only interested in the classification task.

CNN architectures can be broadly classified as shallow or deep, each suited to different tasks and datasets.

In this paper, we are inspired in the first step by a shallow architecture introduced in [16,33]. This relatively simple CNN architecture is composed of two convolution layers, two max-pooling layers, and three FC layers.

There are also many complex architectures that are widely used in the field of optical images due to the abundance of annotated data in this domain. This allows DNNs applied to this domain to have very good performances. We can cite, for example, the following very deep architectures: VGG [34], ResNet [35], Xception [36], DenseNet [37], MobileNetV2 [38], EfficientNet [39], and Siamese and RasNet Neural Networks [13,16].

#### 2.1.1. Description of CNN Architecture Adopted

In this section, the proposed CNN architecture is highlighted. The design aims to balance computational efficiency and overfitting prevention. The proposed architecture includes three convolutional (CONV) layers and three fully connected layers, $n_{fc}$ = 3. Input data comprise a tensor $R\in R^{N_{R}\times N_{s}\times N_{s}}$, where $N_{R}$ = 1 and $N_{s}$ = 128. The proposed CNN includes four steps in convolutional layers to extract features before classification. These steps comprise the following:

- Zero-padding step: Ensures no information is lost at the borders during convolution. If ($Z_{1}$,$Z_{2}$) $\in N^{2}$ denotes the number of zeros added to the last two tensor dimensions, the zero-padding step constructs the tensor $R_{\mathrm{pad}}=\left(r_{t,s_{1},s_{2}}^{pad}\right)\in R^{N_{R}\times \left(N_{s}+Z_{1}\right)\times \left(N_{s}+Z_{2}\right)}$.
- Convolutional step: Extracts features by applying filters to the input tensor. Each filter moves across the tensor with defined strides, producing an output that highlights key spatial patterns. The process is parameterized by the number, size, and strides of the filters, optimizing feature extraction for the classification task.
  If *K* denotes the number of filters, $W_{k}=\left(w_{t,u_{1},u_{2}}^{k}\right)\in R^{N_{R}\times U_{1}\times U_{2}}$ represents the $k^{\mathrm{th}}$ filter where ($U_{1}$,$U_{2}$) $\in {N^{*}}^{2}$ is the size of all filters, and ($a_{1}$,$a_{2}$) $\in {N^{*}}^{2}$ denotes the strides of filters along the last two dimensions, then the output of the convolutional step is mathematically given by
  (1) $$r_{k,s_{1c},s_{2c}}^{conv}=\sum_{t=1}^{T}\sum_{u_{1}=1}^{U_{1}}\sum_{u_{2}=1}^{U_{2}}w_{t,u_{1},u_{2}}^{k}r_{t,a_{1}\beta_{c}+u_{1},a_{2}\omega_{c}+u_{2}}^{pad}\;;$$
  where
  $$\begin{array}{cc} \beta_{c} & =s_{1c}-1\\ \omega_{c} & =s_{2c}-1\\ s_{1c} & \in [1,2,\cdots ,N_{conv,1}]\\ s_{2c} & \in [1,2,\cdots ,N_{conv,2}]\\ k & \in [1,2,\cdots ,K]\\ N_{conv,1} & =\mathrm{floor}\left(\frac{N_{s}+Z_{1}-U_{1}}{a_{1}}\right)+1,\;and\\ N_{conv,2} & =\mathrm{floor}\left(\frac{N_{s}+Z_{2}-U_{2}}{a_{2}}\right)+1 \end{array}$$
  Then, the resulting tensor is given by
  (2) $$R_{\mathrm{conv}}=\left(r_{k,s_{1c},s_{2c}}^{conv}\right)\in R^{K\times N_{conv,1}\times N_{conv,2}}$$
  An activation function is then applied to this tensor. The Rectified Linear Unit (ReLU) activation function is used.
- Max-pooling step: Downsamples the feature maps by retaining the highest value within a defined window, reducing dimensionality while preserving the most significant features. This process enhances computational efficiency and focuses on dominant spatial patterns.
  If ($V_{1}$,$V_{2}$) $\in {N^{*}}^{2}$ is the size of the max-pooling window and ($b_{1}$,$b_{2}$) $\in {N^{*}}^{2}$ are its strides along the last two dimensions, respectively, the output of max-pooling applied on the activated CONV tensor
  (3) $$R_{\mathrm{conv}.\mathrm{act}}=\left(r_{k,s_{1c},s_{2c}}^{conv.act}\right)\in R^{K\times N_{conv,1}\times N_{conv,2}}$$
  can be expressed by
  (4) $$r_{k,s_{1mp},s_{2mp}}^{output}=\max_{v_{1}=1}^{V_{1}}\max_{v_{2}=1}^{V_{2}}\left(r_{k,b_{1}\beta_{mp}+v_{1},b_{2}\omega_{mp}+v_{2}}^{conv.act}\right)\;;$$
  where
  $$\begin{array}{cc} k & \in [1,2,\cdots ,K]\\ \beta_{mp} & =s_{1mp}-1\\ \omega_{mp} & =s_{2mp}-1\\ s_{1mp} & \in [1,2,\cdots ,N_{maxp,1}]\\ s_{2mp} & \in [1,2,\cdots ,N_{maxp,2}]\\ N_{maxp,1} & =\lfloor \frac{N_{conv,1}-V_{1}}{b_{1}}\rfloor +1,\;and\\ N_{maxp,2} & =\lfloor \frac{N_{conv,2}-V_{2}}{b_{2}}\rfloor +1 \end{array}$$
- Dropout step: Mitigates overfitting by randomly setting a fraction of the tensor’s elements to zero during training. This regularization technique reduces the network’s reliance on specific neurons, improving its generalization to unseen data.

The structure of the dense layers and feature extraction influence the classification performance, and the choice between deep or shallow architectures depends on the characteristics of the dataset and the application tasks.

The used datasets represent shallow datasets, i.e., each has a low number of samples per class relative to the size of dataset required to train deep learning-based methods. In fact, the FUSAR-Ship, MSTAR, and OpenSARShip datasets only have 580, 275, and 224 training samples per class on average, respectively.

Basha et al. [40] reported that shallow models perform better than deeper CNNs on shallower datasets. On the basis of this observation, the number of FC layers is fixed to three.

Another observation reported in [40] is that deeper architectures require fewer neurons in FC layers in order to achieve better performance, regardless of the size and type of the dataset. Therefore, to reduce the output, FC layers decrease in terms of the number of neurons used, that is, if $N_{i}$ and $N_{out}$ are, respectively, the number of neurons in the *i*th and the last FC layers, where i∈[1,2,3], then $N_{1}>N_{2}>N_{3}>N_{out}$. A parameter $N\in N^{*}$ is defined to parameterize the number of neurons in the first three FC layers, which are determined as $N_{1}$ = *N*, $N_{2}$ = $\frac{3N}{4}$, and $N_{3}$ = $\frac{N}{2}$. $N_{out}$ = *C* is the number of targeted classes.

The output of the FC layers depends on the weight between the neurons and the activation function. Let $L_{i}$ and $L_{j}$ represent two fully connected layers, where the neurons in $L_{i}$ are fully connected to those in $L_{j}$ and ($N_{i},N_{j}$) $\in {N^{*}}^{2}$ denote their respective number of neurons. If ($y_{n_{i}}$,$w_{n_{i}}^{n_{j}}$) $\in R^{2}$ represent the output of the $n_{i}$^th^ neuron in layer $L_{i}$ and the connection weight between this neuron and the $n_{j}$^th^ neuron in layer $L_{j}$, the input value of the $n_{j}$^th^ neuron is calculated as follows:

(5) $$x_{n_{j}}=\sum_{n_{i}=1}^{N_{i}}w_{n_{i}}^{n_{j}}y_{n_{i}}$$

where $n_{j}\in \left[1,\ldots ,N_{j}\right]$.

Next, the resulting value is processed by a ReLU activation function. In each of the first two FC layers, half of the ReLU activation outputs are set to zero by a dropout before being passed to the next FC layer. No dropout is applied in the last two layers, as the information in these these layers is crucial for classification. Table 1 presents the initial parameter configuration for the proposed CNN architecture, where the bolded settings are fixed and the others are subject to a model selection procedure.

The proposed system minimizes the Cross-Entropy (CE) loss between the truth label classes of training images and their estimates provided by the CNN output layer. The optimization of the weight values is performed using backpropagation and an Adam optimizer with an initial learning rate of 10^−4^ that was decreased by a factor of 0.2 whenever the validation loss stopped improving for more than 10 epochs (adaptive decay). During each training process, the training dataset is decomposed into batches of 32 images. For each epoch, the metrics (loss and accuracy) in the validation set are calculated, and weights are saved if a lower value of the loss is obtained. Early stopping is applied to terminate the training if the validation loss does not decrease after a set number of epochs, which is fixed at 15 iterations in this study.

#### 2.1.2. Model Selection for the CNN (Methodology)

This subsection discusses the model selection procedure of the proposed CNN architecture. In this part, the variations of some parameters according to permanent settings shown in Table 1 are highlighted.

We aim to find the best numbers and size of convolutional kernels, as well as the best number of neurons in FC layers resulting in the best possible performance on the validation set.

**Figure 1:**
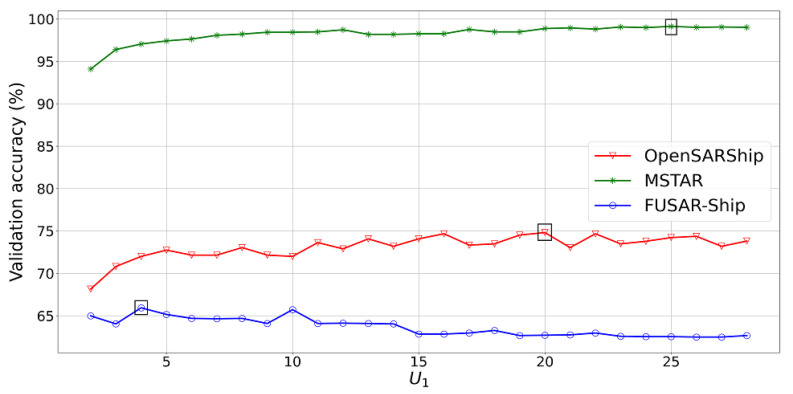
Classification performance of the CNN on the validation set in the function of the squared size of convolutional kernels ($U_{1}$,$U_{2}$) with $U_{1}$ = $U_{2}$ and ($K_{1}$,$K_{2}$,$K_{3}$) = (64,64,128) and *N* = 256.

To ease this analysis, we consider architectures where the size of convolutional kernels is the same for all CONV layers, i.e., ${\left(U_{1},U_{2}\right)}_{i}$ = ($U_{1}$,$U_{2}$), i∈[1,2,3]. Squared kernels are tested with sizes ranging from (2,2) to (28,28) (Figure 1). So, the optimal parameters for ($U_{1}$,$U_{2}$) are (4,4) for FUSAR-Ship, near (20,20) for OpenSARShip, and near (25,25) for MSTAR.

We then explore variations where the number of convolutional kernels in the last CONV layer is twice that of the first layer, i.e., $K_{3}=2K_{1}$, and where the number of kernels in the second CONV layer is set equal to one of the other layers, i.e., $K_{2}=K_{1}$ or $K_{2}=K_{3}$. High numbers of kernels ($K_{i}>512$, i∈[1,2,3]) are not tested to avoid excessively long training times and to reduce the risk of overfitting (Table 2).

With the number of neurons in the FC layers defined by the parameter *N*, we explore different variations of this parameter (Table 3).

Carefully selecting the width of the CNN is crucial for achieving optimal performance. At this stage, the number and size of kernels are set to their optimal values. The output tensor from the final CONV layer has dimensions $N_{f}=2\times 2\times K_{3}$. Given the decreasing number of neurons in the FC layers, the following conditions must be met: $N_{1}<N_{f}$, and $N_{\left(n_{fc}-1\right)}>N_{out}$. These conditions restrict the parameter *N* in the interval [$N_{f}$,2$N_{out}$]. We then choose a set of values uniformly distributed over this interval to be evaluated as *N*.

The optimal parameters for the proposed CNN model for each of the considered datasets are shown in Table 4. Note that better prediction performance is found for deeper datasets, e.g., OpenSARShip and MSTAR. These parameters are used in the CNN-LSTM hybrid network architecture (Section 3.3).

## 3. Recurrent Neural Networks (RNNs)

### 3.1. Description of RNN

An RNN is a DNN that possesses recurrent connections which give the ability to map an input sequence to an output sequence while at each step taking the information of previous steps into account.

Two major difficulties have been identified when training an RNN: the vanishing and exploding gradient problems [41]. When the gradient vanishes, the network basically stops learning, and when it explodes, it can cause weights to oscillate between different values [3]. These two phenomena have a similar origin. When applying backpropagation on DNNs, one must concatenate more and more multiplications of activations as it goes back in the network. These activations are bounded, in the case of the sigmoid function, between [0, 1], and this causes the gradient signal to vanish. This problem appears in every DNN, although simple solutions have been found for feed forward networks, such as the use of the ReLU [42] instead of the sigmoid function and the introduction of skip connections in so-called Residual Networks (ResNets) [35].

### 3.2. Long Short-Term Memory Network (LSTM)

**Figure 2:**
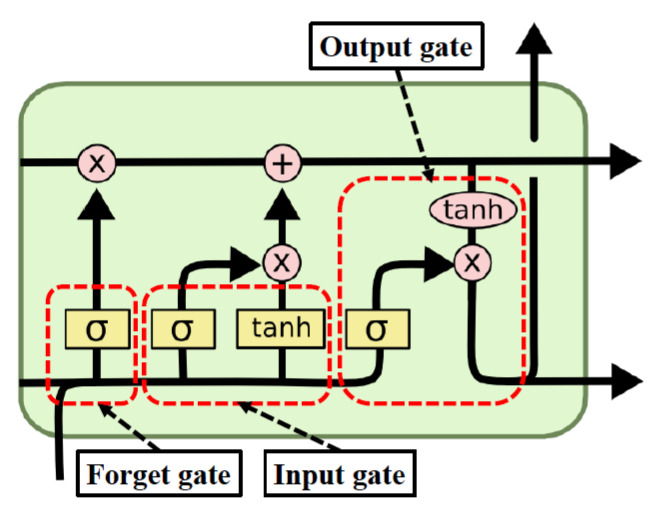
The repeating LSTM module [43].

Hochreiter and Schmidhuber [4] proposed a way around the vanishing/exploding gradient problems to allow RNNs to learn long-term dependencies by introducing a gating mechanism: the long short-term memory (LSTM) (cf. Figure 2).

The first step in an LSTM is to decide what information we are going to throw away from the cell state. This decision is made by a sigmoid layer called the forget gate layer. It looks at $h_{t-1}$ and $x_{t}$ and outputs a number between 0 (completely forget) and 1 (completely keep) for each number in the cell state $C_{t-1}$:

(6) $$f_{t}=\sigma \left(W_{f}\cdot \left[h_{t-1},x_{t}\right]+b_{f}\right)$$

The subsequent step involves determining the new information to be stored in the cell state. Initially, a sigmoid layer, known as the input gate layer, determines which values need updating. Following this, a tanh layer generates a vector of new potential candidate values, ${\tilde{C}}_{t}$, that might be incorporated into the state:

(7) $$\begin{array}{cc} i_{t} & =\sigma (W_{i}\cdot \left[h_{t-1},x_{t}\right]+b_{i})\\ {\tilde{C}}_{t} & =\tan h(W_{C}\cdot \left[h_{t-1},x_{t}\right]+b_{C}) \end{array}$$

Afterwards, the old cell state, $C_{t-1}$, is updated into the new cell state $C_{t}$. The old state is multiplied by $f_{t}$, then the new scaled candidate values $i_{t}{\tilde{C}}_{t}$ are added:

(8) $$C_{t}=f_{t}\circ C_{t-1}+i_{t}\circ {\tilde{C}}_{t}$$

Finally, the output will be a filtered version of the cell state. Initially, a sigmoid layer determines which portions of the cell state will be sent as output. Following this, the cell state undergoes a tanh transformation (to limit the values between −1 and 1) and is then multiplied by the output of the sigmoid gate, ensuring that only the chosen parts are output:

(9) $$\begin{array}{cc} o_{t} & =\sigma (W_{o}\cdot \left[h_{t-1},x_{t}\right]+b_{o})\\ h_{t} & =o_{t}\circ \tan h\left(C_{t}\right) \end{array}$$

### 3.3. Combined CNN-LSTM Hybrid Network Adopted

#### 3.3.1. Description of CNN-LSTM Architecture Adopted

In this work, a hybrid method was developed to classify ships using SAR images. The structure of this architecture is conceived by combining CNN and LSTM networks where a CNN is used to extract the complex features from input images and an LSTM is used as a classifier.

**Figure 3:**
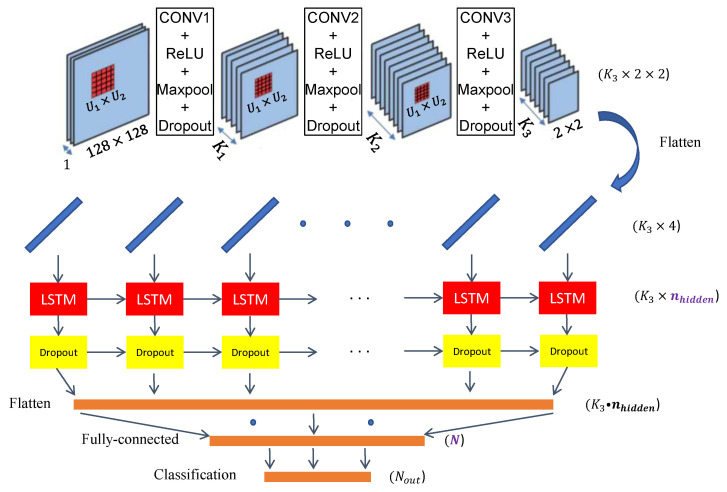
Illustration of the CNN-LSTM network for SAR image classification.

Figure 3 illustrates the proposed combined network for SAR image classification. Each CONV layer has the same steps described in Section 2. The convolutional kernel is extracted by multiplying the superposition matrix in all convolution operations. In the last part of the architecture, the function map is flattened into $K_{3}$ vectors of length 2×2 transferred to the LSTM layer to extract dependency information in terms of kernel ranking. This advanced RNN layer consists of a multiple-input multiple-output (MIMO) structure where several flattened convolutional kernels with multiple ranks, analogous to time steps in the common use of LSTMs, are fed to the network to obtain multiple output features. The output of the LSTM layer is $K_{3}$ vectors of length $n_{hidden}$, where $n_{hidden}$ is the size of the hidden state, which will be optimized during model selection since the performance of such a network depends on this hyperparameter. In total, 50% dropout layers are applied to the outputs of hidden layers, and then an FC layer with *N* neurons connects the hidden state to the FC layer of the softmax function. Finally, this final FC layer is used to predict into $N_{out}$ categories presented in the given dataset.

The structure of the proposed architecture is shown in Table 5. Layers 1–6 of the network are convolutional layers, and layer 7 is the LSTM layer. After the CONV layers, the output shape is found ($K_{3}$, 2, 2) per image. The input size of the LSTM layer is ($K_{3}$, 4). After analyzing LSTM characteristics, the architecture finally sorts SAR images through an FC layer and a softmax layer.

#### 3.3.2. Model Selection for the CNN-LSTM (Methodology)

**Figure 4:**
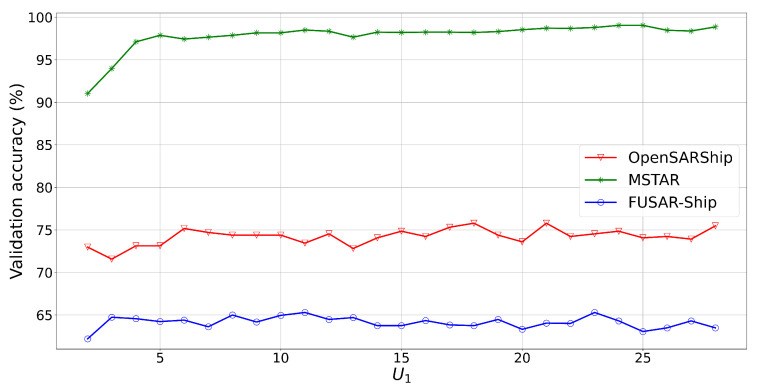
Classification performance of the CNN-LSTM on the validation set in the function of the squared size of convolutional kernels ($U_{1}$,$U_{2}$) with $U_{1}$ = $U_{2}$ and ($K_{1}$,$K_{2}$,$K_{3}$) = (64,64,128), $n_{hidden}$ = 128, and *N* = 128.

To simplify the analysis, the convolutional kernels are assumed to have the same size across all CONV layers. Squared kernels with sizes ranging from (2,2) to (28,28) are tested (Figure 4). So, the optimal parameters for ($U_{1}$,$U_{2}$) are (11,11) for FUSAR-Ship, close to (18,18) for OpenSARShip, and near (24,24) for MSTAR.

In Table 6, we present the accuracy of the model on the validation basis obtained for each dataset. The hyperparameters with the best accuracy are retained.

In Table 7, we varied the size of hidden state ($n_{hidden}$) using the hyperparameters selected in the previous phase. Furthermore, in Table 8, we varied the number of neurons in the FC layer (N) using the hyperparameters selected in the previous phase.

The optimal parameters for the proposed CNN-LSTM network are shown in Table 9 for each dataset. It can be noticed that the optimal CNN-LSTM architectures result in slightly higher prediction accuracies than those of the optimal CNNs, yet this trend has to be verified on the testing dataset.

## 4. Brief Presentation of the SAR Datasets

Unlike optical images in computer vision, which can be easily collected and interpreted, SAR images are much more difficult to annotate due to their complex properties. Several publicly available datasets of SAR images were identified with which to conduct experiments and evaluate ship classification using the proposed architecture. In this section, we briefly present the applied datasets we chose to work on, MSTAR, OpenSARShip, and FUSAR-Ship.

### 4.1. MSTAR Data and Pre-Processing

The MSTAR (Moving and Stationary Target Acquisition and Recognition) database (https://www.sdms.afrl.af.mil/index.php?collection=mstar, accessed on 10 July 2022) [44] contains a set of images collected in 1996 in X band (8–12 GHz) with HH polarization and a resolution of 30 cm. The used acquisition mode is the hyperfine capture or Spotlight, which allows one to have better resolutions because the airborne radar is always directed towards the target during its movement [45].

**Figure 5:**
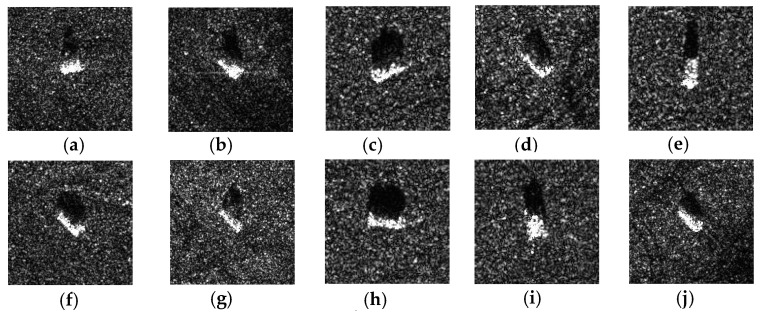
Example presentation in the MSTAR dataset. (**a**) 2S1; (**b**) BMP2; (**c**) BRDM2; (**d**) BTR60; (**e**) BTR70: (**f**) D7; (**g**) T62; (**h**) T72; (**i**) ZIL31; (**j**) ZSU234.

Figure 5 presents an example of SAR images of the different targets. The pixel values of MSTAR data are on a scale of 0 to 255. Therefore, we convert them to floating point numbers and normalize the values by applying a factor of $\frac{1}{255}$. The images of MSTAR are of size 128×128 pixels. The database is provided with an already restored distribution into test and entire training databases. For the training process, we randomly split the entire training database into training and validation subsets with ratios of 80% and 20%, respectively. The validation base allows the monitoring of the quality of training and thus serves as a good indicator for hyperparameter tuning for model selection.

The MSTAR database is a publicly available dataset of synthetic aperture radar (SAR) images. This benchmark is used for automatic target recognition (ATR) tasks. It consists of 5165 images of 10 classes that correspond to different ground targets, such as trucks, tanks, and cars. The numbers of images per class for entire training and test sets are summarized in Table 10.

### 4.2. OpenSARShip Data and Pre-Processing

The SAR image database OpenSARShip [46] is used in the recent scientific literature for the evaluation of SAR image classification algorithms [33,47]. This dataset is composed of SAR image chips of ships, extracted from images produced by Sentinel-1 satellites. These vignettes are derived from two types of products: SLC and GRD. For each of these products, VV and VH polarities are provided, in amplitude only for GRD and in complex for SLC. The corresponding files are provided in different forms: original data, calibrated data, pseudo-color visualization, and grayscale visualization.

The characteristics of the objects in this dataset are also very variable. In particular, the dataset presents a very variable number of instances per class, as well as a great variability of the dimensions and the resolution of the images.

The dataset includes 5673 objects in 68 different classes. The classes correspond to the “Elaborated_type” characteristic present in the metadata of each object provided in the OpenSARShip dataset. Note that different classes have a very small number of instances, which does not allow for efficient deep learning to extract discriminative information that is useful in the generalization phase. To overcome this problem, we retained only the most represented classes of this dataset when using it. This observation is generally noted and adopted in the literature. The authors in [33,47] retained only three classes: respectively, {Bulk Carrier, Container Ship, Tanker} and {Cargo, Bulk Carrier, Container Ship}. In this study, we retained three classes: {Cargo, Bulk Carrier, Container Ship}.

**Figure 6:**
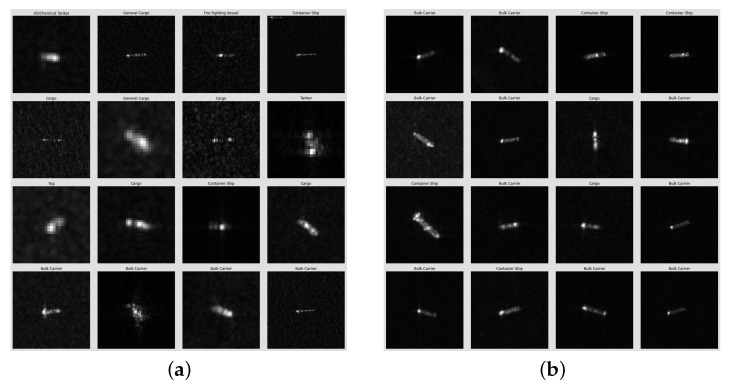
Examples of objects (**a**) from the OpenSARShip dataset and (**b**) from the selected OpenSARShip subpart (VV amplitude).

The SAR images in this dataset also vary greatly in size and resolution, and this difference can greatly complicate the learning task of the neural network (Figure 6). Indeed, objects of the same class but with very different resolutions present different characteristics. It is then more complex for a neural network to extract discriminating characteristics of a class compared to the others. For this reason, the authors in [47] retained images with a minimum size of 70×70 pixels to ensure a minimum resolution when using them. So, we also selected and retained only the targets with the following characteristics:

- Type OD product: GRD.
- Polarization: VV.
- Image size: >70×70, and we resized the images to 128×128 pixels.
- Class: {Cargo, Bulk Carrier, Container Ship}.

In order to perform training, we needed to batch the images, which required images of the same size. We therefore resized the images to 128×128 pixels. Each image was either cropped while keeping the central part or filled with null pixels. Therefore, we did not change the pixel values since no interpolation was performed.

For the evaluation of deep learning algorithms, the data were divided into three subparts: a training base, a validation base, and a test base. We thus obtained the class distribution in the four subsets given by Table 11. The training base allowed the model to learn the deep learning algorithms. The validation base allowed the monitoring of the quality of the training process and thus served as an indicator for hyperparameter tuning. The entire training set, which contains both training and validation sets, was used to re-train the model with the optimal hyperparameters. The test base allowed a prediction performance evaluation independently of the training process and thus allowed us to measure the generalization capability of the trained algorithm.

### 4.3. FUSAR-Ship Data and Pre-Processing

**Figure 7:**
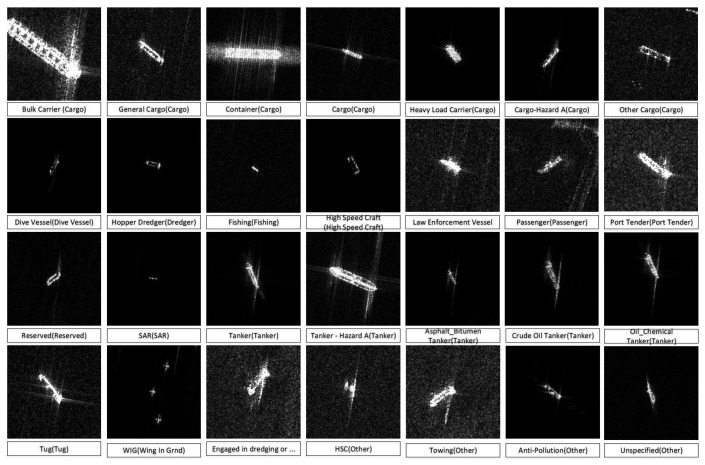
Different categories of ships in FUSAR-Ship [48].

FUSAR-Ship is an open SAR-AIS matchup dataset derived from the Gaofen-3 satellite, backed by the Key Laboratory for Information Science of Electromagnetic Waves (MoE) at Fudan University. Gaofen-3 (GF-3) serves as China’s inaugural civil C-Band fully polarimetric spaceborne synthetic aperture radar (SAR), mainly tasked with oceanic remote sensing and marine monitoring. The FUSAR-Ship dataset was assembled using an automatic SAR-AIS matchup procedure applied to over 100 GF-3 scenes, encompassing a wide range of sea, land, coastal, river, and island environments (Figure 7). It comprises more than 5000 ship image chips with corresponding AIS messages (AIS: automatic identification system) and includes various other maritime targets and background clutter. FUSAR-Ship is designed as a public benchmark dataset for the detection and recognition of ships and marine targets [48].

All image chips are 512×512 pixels and are extracted from the original GF-3 L1A images. The ship is consistently positioned at the center, though the chip might also contain adjacent ships or other items.

In this study, we chose a subset that contains four common ship classes, Cargo, Bulk Carrier, Fishing, and Tanker, from the original dataset. For the evaluation of deep learning algorithms, we proceeded with the same process presented for the two previous datasets. The data were divided into three subparts: a training set, a validation set, and a test set. We thus obtained the class distribution in the four subsets given by Table 12.

## 5. Experiments and Results on SAR Images

The PyTorch open source library was used to implement the proposed solution in Python 3.7. Training was supported by an NVIDIA-SMI 440.82 with CUDA toolkit 10.1.

**Figure 8:**
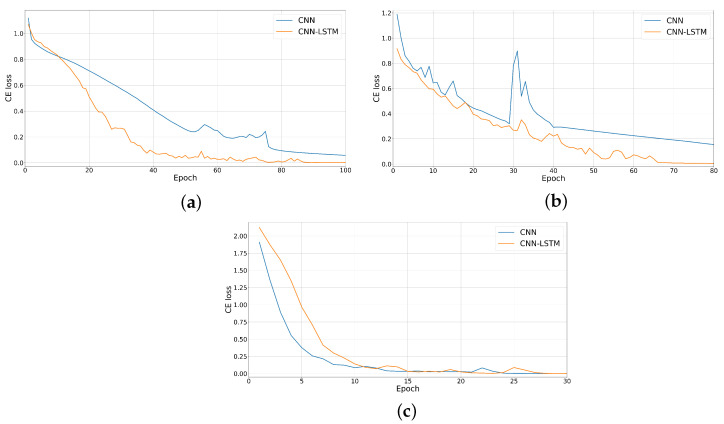
Evolution of the CE loss during training for (**a**) FUSAR-Ship, (**b**) OpenSARShip, and (**c**) MSTAR datasets.

**Figure 9:**
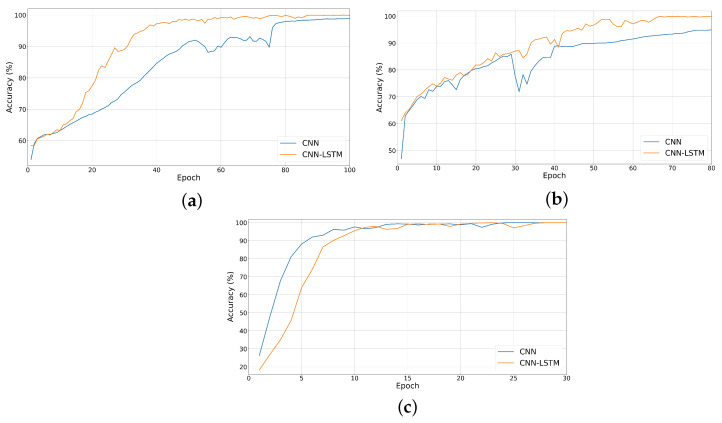
Evolution of classification accuracy during training for (**a**) FUSAR-Ship, (**b**) OpenSARShip, and (**c**) MSTAR datasets.

Figure 8 and Figure 9 show a comparison between the training plots of the proposed networks for each of the FUSAR-Ship, OpenSARShip, and MSTAR datasets. The evolution of CE loss during training is shown in Figure 8, while the evolution of classification accuracy is shown in Figure 9. We can see that for the case of the OpenSARShip dataset, the combined network is more stable while learning and converges faster. In contrast, an opposite trend is observed for the deeper MSTAR dataset. In this case, the proposed CNN converges faster, but it has a stability that is similar to that of the combined network.

By analyzing the results, it is demonstrated that a combination of CNN and LSTM has significant effects on the classification of ships based on the automatic extraction of features from SAR images.

**Figure 10:**
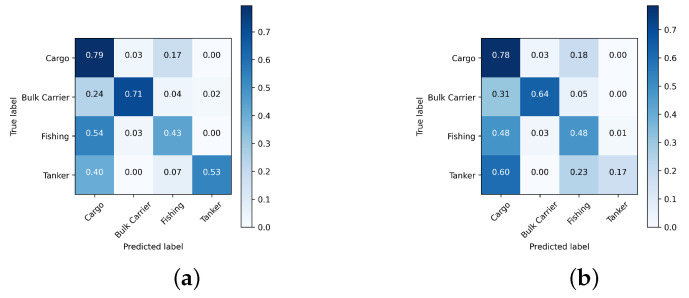
Normalized confusion matrix of the proposed (**a**) CNN and (**b**) CNN-LSTM architectures using FUSAR-Ship dataset.

**Figure 11:**
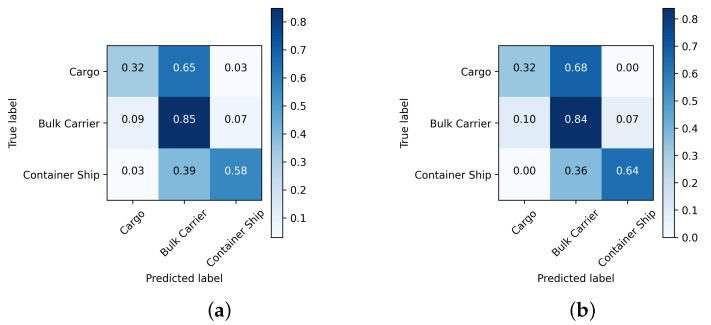
Normalized confusion matrix of the proposed (**a**) CNN and (**b**) CNN-LSTM architectures using OpenSARShip dataset.

**Figure 12:**
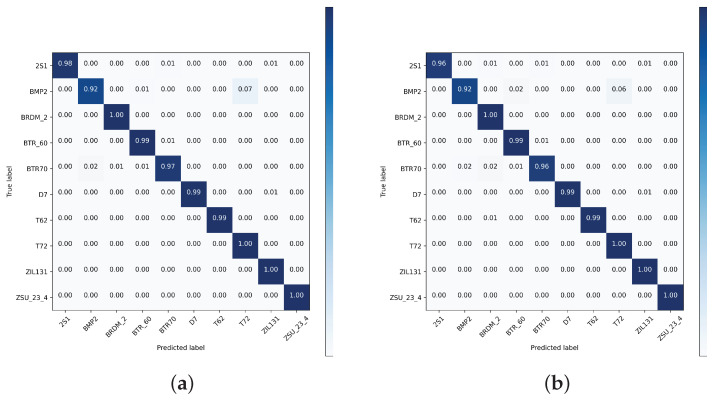
Normalized confusion matrix of the proposed (**a**) CNN and (**b**) CNN-LSTM architectures using MSTAR dataset.

Figure 10, Figure 11 and Figure 12 depict the normalized confusion matrices of the proposed DNNs for ship classification on the FUSAR-Ship, OpenSARShip, and MSTAR testing sets, respectively.

For the OpenSARShip test set, the proposed methods have a relatively good classification performance for the class “Bulk Carrier”, which is the most represented class in the test set, i.e., 62.13% of testing samples. For both networks, there are still cases of misclassification for the least represented classes. It can be seen that for the CNN, 20 of “Cargo” ships were misjudged as “Bulk Carrier”, with an inter-class error of 64.5%, and 13 of the ”Container Ship” images were misjudged as “Bulk Carrier”, with an inter-class error of 39.4%, indicating that both “Cargo” and “Container Ship” are the classifications that can be easily confused with “Bulk Carrier”. This is mainly due to the imbalance between classes in terms of the number of samples and the low number of training images. Overall, the main difference between both networks is that for the hybrid network, the inter-class error is null between the least represented classes, that is, ”Cargo” and ”Container Ship”, in contrast to 3.2% for the proposed CNN.

Regarding the MSTAR dataset, both proposed architectures classify test images with high accuracy. Among ten classes, there are four classes whose images are perfectly classified, and there are, respectively, six and seven classes whose classification accuracy is higher than 99% for the CNN and the CNN-LSTM. For both cases, the class with the relatively low classification accuracy is “BMP2”. Regarding the prediction of images belonging to this class, it is found that the proposed CNN-LSTM network slightly outperforms the competitive CNN network as it has a lesser confusion ratio between “BMP2” and “T72”, i.e., 9% for the combined network in contrast to 6% for the CNN. Hence, the proposed CNN-LSTM system can efficiently classify ship images on a deeper and more balanced dataset.

The comparative study presented in Table 13, Table 14 and Table 15 evaluates the performance of the proposed CNN and CNN-LSTM networks against existing state-of-the-art architectures. This analysis reveals the strengths and limitations of the proposed methods, offering a balanced perspective on their application.

One significant advantage of the proposed methods is their efficiency in training time. The CNN-LSTM network substantially reduces training time compared to both the standalone CNN and other architectures. On the OpenSARShip dataset (Table 14), the CNN-LSTM network completes training in just 132.61 s, whereas VGG16 requires 718.57 s. Such efficiency makes the CNN-LSTM particularly suitable for time-sensitive applications and scenarios with computational constraints. Additionally, the CNN-LSTM achieves a marked reduction in test loss, improving probabilistic class predictions. On the OpenSARShip and MSTAR datasets, the test loss is reduced by 63.93% and 38.78%, respectively, when compared to the standalone CNN (Table 14 and Table 15).

The CNN-LSTM also delivers competitive accuracy, outperforming most existing architectures. For instance, on the OpenSARShip dataset, the CNN-LSTM achieves an accuracy of 70.41%, which is higher than ResNet50 (57.99%) and Xception (65.09%) (Table 14). Furthermore, the proposed methods demonstrate strong adaptability to datasets with fewer and unbalanced instances, such as OpenSARShip, highlighting their robustness in challenging scenarios. The lightweight design of the standalone CNN, with a parameter count as low as 363 k on FUSAR-Ship, adds to its appeal for resource-constrained environments (Table 13).

However, the proposed methods have some limitations. While the CNN-LSTM offers significant efficiency gains, its accuracy improvements over top-performing architectures like VGG16 are modest. On the MSTAR dataset, the CNN-LSTM achieves 98.35% accuracy, only slightly higher than VGG16’s 98.14%. Additionally, both the CNN and CNN-LSTM networks exhibit higher test loss on certain datasets, such as FUSAR-Ship, where their test loss ((4.8501 and 4.1756, respectively) surpasses that of DenseNet121 (3.4620) and Xception (2.8643) (Table 13).

Another drawback is the increased parameter count of the CNN-LSTM compared to the standalone CNN. On the MSTAR dataset, the CNN-LSTM’s parameters reach 59.33 M, significantly more than the standalone CNN’s 31.10M, potentially increasing memory requirements (Table 14). Lastly, while the CNN-LSTM performs well on datasets like OpenSARShip and MSTAR, its variable performance across datasets, such as the higher test loss on FUSAR-Ship, suggests it may not universally outperform existing architectures (Table 13).

In summary, the proposed CNN and CNN-LSTM networks offer significant advantages in efficiency and robustness, particularly for datasets with unbalanced classes or in resource-constrained environments. However, their marginal accuracy improvements and variability in performance across datasets indicate that further refinements may be necessary to achieve consistent superiority over state-of-the-art architectures. These methods remain a promising step forward in designing efficient and adaptable neural networks.

## 6. Conclusions

This study addresses the challenges of using deep neural networks (DNNs) for target classification in synthetic aperture radar (SAR) images, particularly with limited labeled data. By balancing complexity and performance, we proposed a CNN architecture and evaluated it on the OpenSARShip, MSTAR, and FUSAR-Ship datasets, focusing on supervised learning with minimal annotated data. Key hyperparameters were optimized through a validation-based model selection process, ensuring robust generalization. We further proposed replacing dense layers with LSTM layers for convolutional feature classification. This combination enhanced both classification accuracy and training efficiency. Comparative analysis demonstrated that our model offers competitive performance while maintaining lower computational costs compared to state-of-the-art architectures commonly used in optical image processing.

Looking ahead, future studies could explore integrating shallow DNNs with attention mechanisms [26] to focus selectively on relevant image regions, thereby improving classification accuracy. Additionally, including advanced techniques such as dilated convolutions—including Hybrid Dilated CNNs (HDCs) [49], mixed convolutional kernels [11], and multi-dilated convolutional blocks—could further enhance feature extraction and reduce reliance on pooling operations [50].

Unsupervised learning represents another promising avenue, enabling the use of large unannotated datasets to independently learn essential image characteristics. Expanding annotated datasets through Generative Adversarial Networks (GANs) or other data augmentation methods could also significantly improve performance. These perspectives hold potential for advancing ship classification and other remote sensing applications.

## Figures and Tables

**Table 1:** Proposed CNN architecture for ship classification.

CNN Layer	Layer Steps	Parameters
Input		$R\in R^{N_{R}\times N_{s}\times N_{s}}$
CONV #1	Zero padding 2D Conv 2D Max-pooling 2D Dropout	${\left(Z_{1},Z_{2}\right)}_{1}$ = (1,1) $K_{1}$ = 64, ${\left(U_{1},U_{2}\right)}_{1}$ = (2,2) ${\left(a_{1},a_{2}\right)}_{1}$ = (1,1) act = ’ReLU’ ${\left(V_{1},V_{2}\right)}_{1}$ = (4,4), ${\left(b_{1},b_{2}\right)}_{1}$ = (4,4) 25%
CONV #2	Zero padding 2D Conv 2D Max-pooling 2D Dropout	${\left(Z_{1},Z_{2}\right)}_{2}$ = (1,1) $K_{2}$ = 64, ${\left(U_{1},U_{2}\right)}_{2}$ = (2,2) ${\left(a_{1},a_{2}\right)}_{2}$ = (1,1) act = ’ReLU’ ${\left(V_{1},V_{2}\right)}_{2}$ = (4,4), ${\left(b_{1},b_{2}\right)}_{2}$ = (4,4) 25%
CONV #3	Zero padding 2D Conv 2D Max-pooling 2D Dropout	${\left(Z_{1},Z_{2}\right)}_{3}$ = (1,1) $K_{3}$ = 128, ${\left(U_{1},U_{2}\right)}_{3}$ = (2,2) ${\left(a_{1},a_{2}\right)}_{3}$ = (1,1) act = ’ReLU’ ${\left(V_{1},V_{2}\right)}_{3}$ = (4,4), ${\left(b_{1},b_{2}\right)}_{3}$ = (4,4) 25%
FC #1	Dense Dropout	$N_{1}$ = *N*, act = ’ReLU’ 50%
FC #2	Dense Dropout	$N_{2}$ = $\frac{N}{2}$, act = ’ReLU’ 50%
Output	Dense	$N_{3}$ = $N_{out}$ = *C*, act = ’identity’

**Table 2:** Validation performance of the CNN for variations in the numbers of kernels in CONV layers with optimized values of ($U_{1}$, $U_{2}$) and N=256.

($K_{1}$, $K_{2}$, $K_{3}$)	Validation Accuracy (%)		
	FUSAR-Ship	OpenSARShip	MSTAR
(32,32,64)	64.95	73.04	97.55
(32,64,64)	65.03	73.19	97.92
(64,64,128)	**65.94**	74.81	97.96
(64,128,128)	65.51	**75.56**	98.65
(128,128,256)	65.03	74.07	98.72
(128,256,256)	63.57	73.93	98.69
(256,256,512)	64.34	74.07	98.65
(256,512,512)	63.61	74.22	**99.12**

The cells highlighted in bold indicate the highest validation accuracies achieved for each dataset.

**Table 3:** Validation performance of the CNN for variations in FC layer neurons with the optimal values of ($U_{1}$, $U_{2}$) and ($K_{1}$, $K_{2}$, $K_{3}$).

*N*	Validation Accuracy (%)		
	FUSAR-Ship	OpenSARShip	MSTAR
128	64.60	73.48	98.91
256	**65.94**	**75.56**	**99.12**
384	64.82	75.41	98.83
512	64.34	74.22	98.69

The cells highlighted in bold indicate the highest validation accuracies achieved for each dataset.

**Table 4:** Optimal parameters of the proposed CNN for each dataset and validation performances of the optimal architectures.

Dataset	Optimal Parameters			Validation Accuracy (%)
	($U_{1}$,$U_{2}$)	($k_{1}$,$k_{2}$,$k_{3}$)	*N*	
FUSAR-Ship	(4,4)	(64,64,128)	256	65.94
OpenSARShip	(20,20)	(64,128,128)	256	75.56
MSTAR	(25,25)	(128,128,256)	256	99.12

**Table 5:** The full summary of the proposed CNN-LSTM hybrid network.

Layer	Type	Kernel	Kernel Size	Stride	Input Size
1	Convolution2D	$K_{1}$	$U_{1}\times U_{2}$	1	1×128×128
2	Pool	-	4×4	4	$K_{1}\times 128\times 128$
3	Convolution2D	$K_{2}$	$U_{1}\times U_{2}$	1	$K_{1}\times 32\times 32$
4	Pool	-	4×4	4	$K_{2}\times 32\times 32$
5	Convolution2D	$K_{3}$	$U_{1}\times U_{2}$	1	$K_{2}\times 8\times 8$
6	Pool	-	4×4	4	$K_{3}\times 8\times 8$
7	LSTM	-	-	-	$K_{3}\times 4$
8	FC	*N*	-	-	$K_{3}$.$n_{hidden}$
9	Softmax	$N_{out}$	-	-	*N*

**Table 6:** Validation performance of the CNN-LSTM for variations in the numbers of kernels in CONV layers with optimized values of ($U_{1}$, $U_{2}$) and $n_{hidden}=128$, N=256.

($K_{1}$, $K_{2}$, $K_{3}$)	Validation Accuracy (%)		
	FUSAR-Ship	OpenSARShip	MSTAR
(32,32,64)	63.78	74.53	97.72
(32,64,64)	64.04	74.06	98.35
(64,64,128)	**65.29**	**75.78**	99.04
(64,128,128)	63.48	74.84	98.64
(128,128,256)	65.20	74.84	98.90
(128,256,256)	64.82	74.53	**99.15**
(256,256,512)	63.74	74.84	99.01
(256,512,512)	64.17	75.00	99.04

The cells highlighted in bold indicate the highest validation accuracies achieved for each dataset.

**Table 7:** Validation performance of the CNN-LSTM for variations in the size of hidden state ($n_{hidden}$) with the optimal values of ($U_{1}$, $U_{2}$) and ($K_{1}$, $K_{2}$, $K_{3}$) and N=128.

$n_{hidden}$	Validation Accuracy (%)		
	FUSAR-Ship	OpenSARShip	MSTAR
32	65.16	75.31	**99.23**
64	64.52	75.47	99.08
96	63.78	73.91	99.19
128	**65.29**	**75.78**	99.15
160	64.34	74.69	99.15
192	64.69	74.84	98.71

The cells highlighted in bold indicate the highest validation accuracies achieved for each dataset.

**Table 8:** Classification performance of the CNN-LSTM on the validation set in the function of the number of neurons in the FC layer (N), with the optimized ($U_{1}$, $U_{2}$), ($K_{1}$, $K_{2}$, $K_{3}$), and $n_{hidden}$ hyperparameters.

*N*	Validation Accuracy (%)		
	FUSAR-Ship	OpenSARShip	MSTAR
64	64.82	75.32	99.16
128	**65.29**	**75.78**	99.15
184	64.85	75.11	99.34
256	64.94	74.98	99.43
320	64.88	75.21	**99.52**
384	64.91	74.84	99.49

The cells highlighted in bold indicate the highest validation accuracies achieved for each dataset.

**Table 9:** Optimal parameters of the proposed combined CNN-LSTM network for each dataset and validation performances of the optimal architectures.

Dataset	Optimal Parameters				Validation Accuracy (%)
	($U_{1}$,$U_{2}$)	($k_{1}$,$k_{2}$,$k_{3}$)	$n_{hidden}$	*N*	
FUSAR-Ship	(11,11)	(64,64,128)	128	128	65.29
OpenSARShip	(18,18)	(64,64,128)	128	128	75.78
MSTAR	(24,24)	(128,256,256)	32	320	99.52

**Table 10:** The distribution of MSTAR data in the entire training/test database.

Targets	2S1	BMP2	BRDM2	BTR60	BTR70	D7	T62	T72	ZIL131	ZSU234
Entire training	299	233	298	256	233	299	299	232	299	299
Test	274	195	274	195	196	274	273	196	274	274

**Table 11:** The number of instances per class in the three subsets resulting from splitting the selected OpenSARShip data.

	Training	Validation	Entire Training	Test
	80% Entire Training	20% Entire Training	80% Dataset	20% Dataset
Cargo	99	25	124	31
Bulk Carrier	335	84	419	105
Container Ship	104	26	130	33

**Table 12:** The number of instances per class in the four subsets resulting from splitting the selected FUSAR-ships dataset.

	Training	Validation	Entire Training	Test
	80% Entire Training	20% Entire Training	80% Dataset	20% Dataset
Cargo	1083	271	1354	339
Bulk Carrier	174	44	218	55
Fishing	502	126	628	157
Tanker	94	24	118	30

**Table 13:** Performance comparison of the proposed CNN and CNN-LSTM networks with existing systems on FUSAR-Ship dataset.

Architecture	Number of	Training	Number	Test Loss	Test
	Parameters	Time (s)	of Epochs		Accuracy (%)
VGG16	134.28M	711.18	99	4.3436	65.23
ResNet50	23.52M	4602.78	763	3.8623	67.99
Xception	20.82M	2822.80	505	2.8643	67.13
DenseNet121	6.96M	5250.83	388	3.4620	71.08
EfficientNetB0	4.01M	1178.61	131	2.3526	61.45
MobileNetV2	2.23M	1090.02	250	2.9414	57.31
Proposed CNN	363k	3447.87	4109	4.8501	67.47
Proposed CNN-LSTM	3.66M	377.54	163	4.1756	65.58

**Table 14:** Performance comparison of the proposed CNN and CNN-LSTM networks with existing systems on OpenSARShip dataset.

Architecture	Number of	Training	Number	Test Loss	Test
	Parameters	Time (s)	of Epochs		Accuracy (%)
VGG16	134.27M	718.57	270	**2.0185**	72.19
ResNet50	23.51M	3502.18	1854	5.8233	57.99
Xception	20.81M	1958.26	978	4.1148	65.09
DenseNet121	6.96M	5578.21	1447	8.4319	56.80
EfficientNetB0	4.01M	254.74	111	2.9316	52.07
MobileNetV2	2.23M	621.78	398	2.5883	56.21
Proposed CNN	10.02M	1375.10	1642	6.9658	69.82
Proposed CNN-LSTM	6.17M	132.61	166	2.5124	70.41

**Table 15:** Performance comparison of the proposed CNN and CNN-LSTM networks with existing systems on MSTAR dataset.

Architecture	Number of	Training	Number	Test Loss	Test
	Parameters	Time (s)	of Epochs		Accuracy (%)
VGG16	138.30M	1073.35	132	0.1532	98.14
ResNet50	23.53M	2902.83	425	0.2698	95.67
Xception	20.83M	3397.96	504	0.1836	95.34
DenseNet121	6.96M	11188.50	731	0.1543	97.77
EfficientNetB0	4.02M	1541.87	155	0.5745	86.85
MobileNetV2	2.24M	1671.27	275	4.2541	40.74
Proposed CNN	31.10M	1880.30	289	0.1239	98.52
Proposed CNN-LSTM	59.33M	686.19	74	0.0910	98.35

## Data Availability

We used in this study three publicly available datasets: MSTAR, OpenSARShip, and FUSAR-Ship. The MSTAR dataset can be found at https://www.sdms.afrl.af.mil/index.php?collection=mstar (accessed on 3 July 2022). The OpenSARShip dataset is available at https://opensar.sjtu.edu.cn/DataAndCodes.html (accessed on 3 July 2022). The FUSAR-ship dataset can be downloaded at https://drive.usercontent.google.com/download?id=1SOEMud9oUq69gxbfcBkOvtUkZ3LWEpZJ&export=download&authuser=0, last accessed on 6 December 2024.

## Abbreviations

The following abbreviations are used in this manuscript:

CNN	Convolutional neural network
LSTM	Long short-term memory
MS-CNN	Multi-Stream CNN
Conv-BiLSTM	Convolutional bidirectional long short-term memory
CBLPN	Conv-BiLSTM Prototypical Network
SAR	Synthetic aperture radar
MSTAR	Moving and Stationary Target Acquisition and Recognition
AIS	Automatic identification system
DNN	Deep neural network
FC	Fully connected
CE	Cross Entropy
GPU	Graphics processing unit
FPGA	Field-Programmable Gate Array
ASIC	Application-Specific Integrated Circuit
RNN	Recurrent neural network
GRD	Ground Range Detected
SLC	Single Look Complex
VV	Vertical–Vertical polarization
VH	Vertical–Horizontal polarization
HDC	Hybrid Dilated CNN
GAN	Generative Adversarial Network
ReLU	Rectified Linear Unit
MIMO	Multiple input multiple output
IT	Inferotemporal Cortex
RGC	Retinal Ganglion Cells
LGN	Lateral Geniculate Nucleus
IoT	Internet of Things
CSI	Channel State Information
RF	Radio Frequency
SSID	Service Set Identifier
BPTT	Backpropagation Through Time
HOG	Histogram of Oriented Gradients
GRSS	Geoscience and Remote Sensing Society
ATR	Automatic target recognition
CUDA	Compute Unified Device Architecture
SMI	System Management Interface
VGG	Visual Geometry Group
ResNet	Residual Network
Xception	Extreme Inception
DenseNet	Densely Connected Convolutional Networks
EfficientNet	Efficient Network
MobileNet	Mobile Network
FUSAR-Ship	Fudan University SAR-Ship
HH	Horizontal–Horizontal polarization
